# Incidental Finding of Gallbladder Torsion During Laparoscopic Cholecystectomy

**DOI:** 10.7759/cureus.18058

**Published:** 2021-09-17

**Authors:** Stephen Doxey, Perwaiz Nawabi, Corey Pagnotta, Sally Smith, Charles Harper, Joshua Gazzetta

**Affiliations:** 1 School of Medicine, Kansas City University of Medicine and Biosciences, Kansas City, USA; 2 Department of General Surgery, St. Mary's Medical Center, Blue Springs, USA

**Keywords:** gallbladder torsion, gallbladder volvulus, laparoscopy, laparoscopic cholecystectomy, cholecystitis, acute cholecystitis, chronic cholecystitis, elderly, detorsion, biliary surgery

## Abstract

Laparoscopic cholecystectomy is a common general surgery procedure, with over a million laparoscopic cholecystectomies performed in the United States annually. A rare presentation, which may be encountered incidentally during surgery, is torsion of the gallbladder. Gallbladder torsion is encountered in 0.01% of all patients with acute cholecystitis. It should be considered in the differential diagnosis of elderly female patients presenting with symptoms of acute or chronic cholecystitis.

In this case report, we discuss the incidental finding of gallbladder torsion during laparoscopic cholecystectomy in an 82-year-old female admitted to the hospital with symptoms of cholecystitis. Preoperative CT imaging revealed a chronic, large hiatal hernia and a dilated gallbladder containing heterogeneous densities, possibly related to sludge. During the operation, a necrotic, torsed gallbladder and long cystic duct were found. A laparoscopic cholecystectomy was performed and the remainder of the patient’s hospital course was uncomplicated.

Intraoperatively, our patient was found to have torsion of the gallbladder. Preoperative lab values revealed mild hyponatremia, hypokalemia, and hypochloremia with normal liver enzymes, bilirubin, and alkaline phosphatase levels. This is consistent with documented cases, as typically the biliary tree is not obstructed. Additionally, preoperative imaging rarely reveals the diagnosis. Prompt detorsion and cholecystectomy should be performed to prevent gangrene and perforation.

Gallbladder torsion can result in perforation if not quickly identified and treated. We recommend prompt laparoscopic detorsion and cholecystectomy to prevent perforation.

## Introduction

Laparoscopic cholecystectomy is a common general surgery procedure, with over a million laparoscopic cholecystectomies performed in the United States annually [1]. A rare and often incidental presentation, encountered in 0.01% of all patients with acute cholecystitis, is torsion of the gallbladder [2]. This uncommon pathology was first described in 1898 [3]. Since the first discovery, over 500 cases have been reported [4]. The incidence of gallbladder torsion has increased since the beginning of the 21st century, possibly due to increased life expectancy [5]. To date, gallbladder torsion accounts for one out of every 365,520 hospital admissions [5].

Gallbladder torsion is the rotation of the gallbladder along its long axis, causing vascular compromise due to ischemic compression of the cystic artery. Torsion is classified as complete, which is ≥180 degrees of rotation, and incomplete, <180 degrees of rotation. The occlusion of the cystic artery due to torsion can further lead to transmural necrosis of the gallbladder [6].

The etiology of gallbladder torsion is not entirely clear, but there are two common predisposing anatomical factors: a mesentery that supports only the cystic duct and artery, and a wide mesentery. These variations allow the gallbladder to float freely from the liver bed so it can rotate about the cystic duct and artery. Additionally, liver atrophy and loss of visceral fat due to aging can lead to an acquired anatomical predisposition to gallbladder volvulus. This may help explain why the highest incidence of gallbladder torsion occurs in the elderly population, with 85% of cases occurring in people between the ages of 60 and 80 years old. Adult women are reportedly more affected than men, with a 3:1 female to male ratio [7].

Preoperative diagnosis is difficult and intraoperative discovery is more common. One report states that 25% of gallbladder torsions are diagnosed preoperatively [6]. Due to the high number of laparoscopic cholecystectomies performed yearly and the 6% mortality rate associated with gallbladder torsion, surgeons should be familiar with this gallbladder pathology [6]. Here we report the case of gallbladder torsion with transmural necrosis and chronic cholecystitis in an elderly woman.

## Case presentation

**Figure 1:**
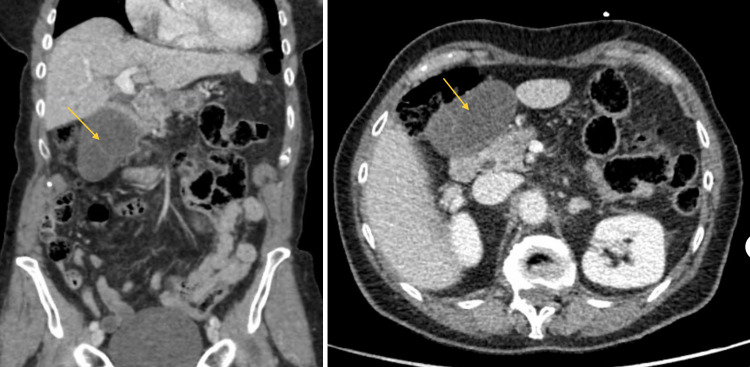
CT abdomen/pelvis. CT abdomen/pelvis revealed a dilated common bile duct and central biliary tree. The gallbladder was found to be dilated, redundant, and exhibited heterogeneous density possibly related to sludge. Gold arrows indicate the gallbladder.

**Figure 2:**
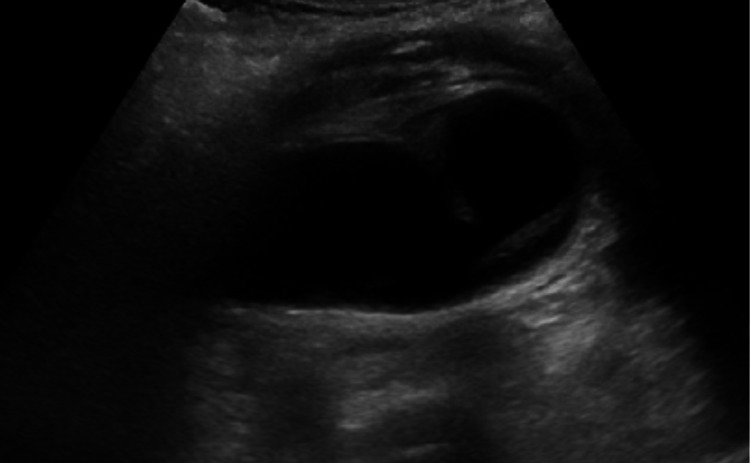
Abdominal ultrasound. Abdominal ultrasound revealed a distended gallbladder with possible internal debris and suggestion of wall thickening. No stones were visualized.

**Figure 3:**
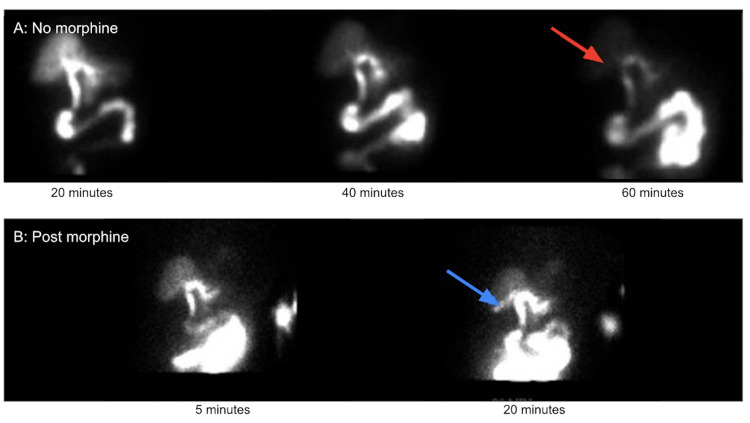
HIDA scan. HIDA scan revealed prompt clearance of tracer from the blood pool and homogeneous distribution throughout the liver. Image A: There was normal emptying into the common bile duct and small bowel. At 60 minutes, the gallbladder was not visualized (red arrow). Image B: The gallbladder was visualized on post morphine imaging at 20 minutes (blue arrow). There was enterogastric reflux during post morphine imaging, which resolved. HIDA: Hepatobiliary iminodiacetic acid.

The patient is an 82-year-old female who underwent laparoscopic cholecystectomy. She initially presented to the hospital with abdominal pain, nausea, and vomiting. Her abdominal pain was localized to her epigastrium and had progressively worsened. The most pronounced symptom was intractable nausea, despite receiving antiemetics. Additional history provided by a family member confirmed that the patient had only been able to tolerate small meals for the past year. The patient’s past medical history included hypertension, hyperlipidemia, and a large hiatal hernia. Physical exam demonstrated a soft, distended abdomen with right upper quadrant tenderness on palpation, without rebound tenderness or guarding. Her chemistry panel revealed mild hyponatremia, hypokalemia, and hypochloremia with normal liver enzymes, bilirubin, and alkaline phosphatase levels. In addition to her electrolyte derangements, she had a WBC count of 10.1 x10^9^ cells/L with a left shift and neutrophil percentage of 81%. CT imaging demonstrated a large hiatal hernia and a dilated gallbladder with heterogeneous densities within the gallbladder, possibly related to sludge. There was a small amount of pericholecystic fluid, extending along the liver surface. The biliary tree was dilated but no calcified gallstones were appreciated (Figure 1). Ultrasound findings demonstrated a distended gallbladder and thickened gallbladder wall measuring 6 mm without cholelithiasis (Figure 2). A hepatobiliary iminodiacetic acid (HIDA) scan was subsequently obtained that showed delayed visualization of the gallbladder (Figure 3). Given the provided history and labs, a presumptive diagnosis of cholecystitis was made. 

**Figure 4:**
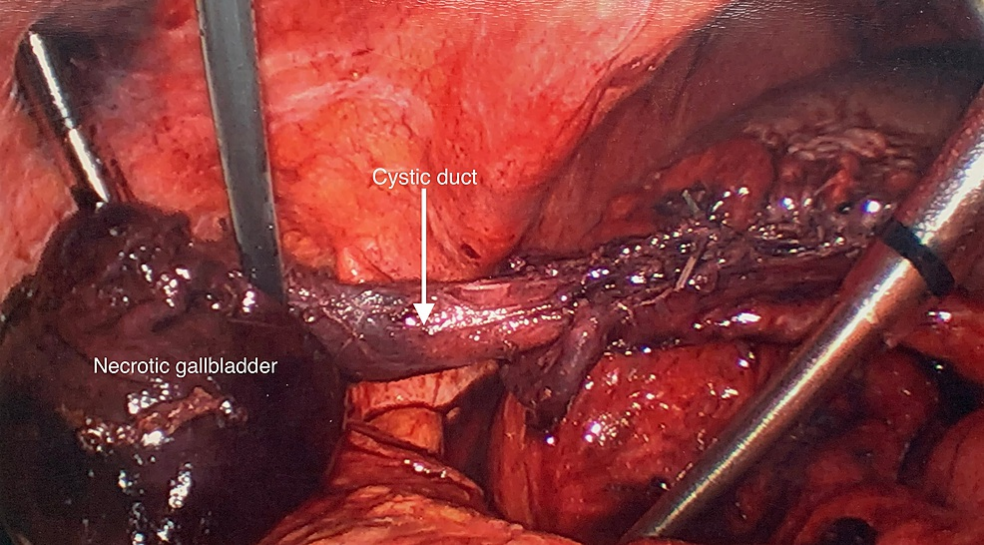
Intraoperative laparoscopic cholecystectomy. Intraoperative findings revealed a long cystic duct and a necrotic gallbladder.

The patient was taken for laparoscopic cholecystectomy on hospital day five, after resolution of electrolyte abnormalities and failure of conservative measures to resolve her symptoms. After Hasson entry and insufflation of the abdomen, laparoscopic visualization of the patient’s right upper quadrant demonstrated a significant amount of inflammation, with the omentum adherent to the abdominal wall and liver. Careful blunt dissection of the omentum away from these structures eventually revealed a large black, mass-like structure. The mass was acutely edematous. Further blunt dissection with a suction irrigator device allowed for the separation of the mass from the liver bed. The mass was found to be a necrotic gallbladder (Figure 4). Once the gallbladder was removed from the surrounding adhesions, it was found to be torsed. Application of a blunt grasper and suction irrigator device successfully detorsed the gallbladder 360 degrees counterclockwise. A very long cystic duct was identified. Clips were applied to the cystic artery and this was divided. The cystic duct was stapled as it was too large to clip. No complications occurred during the procedure. The final surgical pathology revealed chronic cholecystitis with transmural necrosis. The gallbladder was devoid of bile, without calculi, and had wall measurements ranging from 0.1 to 0.6 in thickness with multiple hemorrhagic regions.

## Discussion

Gallbladder torsion typically occurs in elderly females in the sixth to eighth decade of life. Torsion occurs when the gallbladder rotates about the long axis, thus compromising the arterial supply, as well as gallbladder filling and emptying. Preoperative diagnosis of this condition is difficult, but prompt cholecystectomy is indicated as gangrene, perforation, and sepsis may follow. Certain anatomical variations can be associated with a higher incidence of gallbladder torsion. A long cystic duct may be suspended on an abnormally long mesentery, allowing it to hang freely and be susceptible to torsion. Elderly women may be more vulnerable to developing this condition as increased age is correlated with a loss of visceral fat and elasticity, further destabilizing the gallbladder and allowing it to hang freely. In addition to anatomical variations, physiological factors such as peristalsis from the stomach, duodenum, and transverse colon can play a role in initiating torsion. It is unlikely that gallstones play a role in torsion as they have only been documented in approximately 20%-33% of cases. The majority of gallbladder torsions have a clockwise torsion. Incomplete torsions typically mimic the symptoms of pain associated with recurrent biliary colic. Patients with complete torsions typically present with sudden onset, severe right upper quadrant pain, and intractable vomiting. Obstructive cholestasis is not usually observed in laboratory values [8]. 

Imaging such as USG and CT is often obtained yet rarely reveals the diagnosis of gallbladder torsion. CT imaging findings suggestive of possible gallbladder torsion include a fluid collection between the gallbladder and the gallbladder fossa of the liver, a horizontal arrangement of the long axis of the gallbladder, a well-enhanced cystic duct located on the right side of the gallbladder, and signs of inflammation such as edema and gallbladder wall thickening [9]. Magnetic resonance cholangiopancreatography may aid in preoperative diagnosis of gallbladder torsion by showing a V-shaped distortion of the extrahepatic bile ducts due to a twisting of the cystic duct. Additionally, a high-intensity signal on T1-weighted images within the gallbladder wall could indicate a possible infarction [10]. Our patient exhibited a distended gallbladder with possible wall thickening on ultrasound imaging and a dilated and redundant gallbladder on CT imaging. A diagnosis of gallbladder torsion was not suspected based on these imaging findings alone and the definitive diagnosis of gallbladder torsion was not made until surgery. Once identified, diagnosis and treatment via laparoscopy or laparotomy should result in prompt detorsion and cholecystectomy [8].

## Conclusions

Gallbladder torsion is a rare disorder primarily seen in elderly women. Current imaging modalities are unreliable for obtaining a preoperative diagnosis. It is imperative to keep this diagnosis in the differential of elderly patients with signs and symptoms of acute cholecystitis to identify this pathology early and subsequently treat it with cholecystectomy.
